# Localization of Realistic Spatial Patches of Complex Source Activity in MEG and EEG

**DOI:** 10.1109/TNSRE.2025.3622587

**Published:** 2025

**Authors:** Amita Giri, Lukas Hecker, John C. Mosher, Amir Adler, Dimitrios Pantazis

**Affiliations:** Department of Electronics and Communication Engineering, Indian Institute of Technology Roorkee, Roorkee 247667, India, and also with the McGovern Institute for Brain Research, Massachusetts Institute of Technology, Cambridge, MA 02139 USA; Department of Electrical Engineering, Braude College of Engineering, Karmiel 2161002, Israel.; Texas Institute for Restorative Neurotechnologies (TIRN), Department of Neurology, McGovern Medical School, The University of Texas Health Science Center at Houston, Houston, TX 77030 USA.; Department of Electrical Engineering, Braude College of Engineering, Karmiel 2161002, Israel.; McGovern Institute for Brain Research, Massachusetts Institute of Technology, Cambridge, MA 02139 USA.

**Keywords:** MEG, EEG, source localization, alternating projection, extent estimation, rank-2, spatial patches

## Abstract

Accurate localization of neural sources in Magnetoencephalography (MEG) and Electroencephalography (EEG) is essential for advancing clinical and research applications in neuroscience. Traditional approaches like dipole fitting (e.g., MUSIC, RAP-MUSIC) are limited to discrete focal sources, while distributed source imaging methods (e.g., MNE, sLORETA) assume sources distributed across the cortical surface. These methods, however, often fail to capture sources with complex spatial extents, limiting their accuracy in realistic settings. To address these limitations, we introduce PATCH-AP, an enhanced version of the Alternating Projection (AP) method that effectively localizes both discrete and spatially extended sources. We evaluated PATCH-AP against leading source localization methods, including distributed source imaging techniques (MNE, sLORETA), traditional dipole fitting (AP), and recent extended source methods (Convexity-Champagne (CC), FLEX-AP). PATCH-AP consistently outperformed these methods in simulations, achieving lower Earth Mover’s Distance (EMD) scores—a metric indicating closer alignment with the true source distribution. In tests with real MEG data from a face perception task and auditory task, PATCH-AP demonstrated high alignment with the fusiform face area and auditory cortex region. These results highlight PATCH-AP’s potential to enhance source localization accuracy, promising significant advancements in neuroscience research and clinical diagnostics.

## I. Introduction

Magnetoencephalography (MEG) and electroencephalography (EEG) are widely utilized techniques known for their excellent temporal resolution, making them invaluable for capturing dynamic neural activity [1], [2], [3], [4], [5], [6], [7]. These techniques provide key insights into the intricate dynamics of both healthy and neurologically impaired brains. A significant drawback, however, of MEG and EEG is their limited spatial resolution, which presents challenges for accurately localizing neural sources. This difficulty stems from the inherently ill-posed nature of the inverse problem in source localization: the potential neural source locations far outnumber the sensors on or around the scalp, leading to an infinite number of possible source configurations that could explain the recorded signals.

Inverse source localization methods are broadly classified into two categories [8], [9]: distributed source imaging and dipole fitting. Distributed source imaging models assume sources are spread across the entire cortical surface, where source localization involves estimating a density map of these active sources using linear optimization techniques such as the minimum norm estimate (MNE) [10], weighted MNE (WMNE) [11], [12], low-resolution electromagnetic tomography (LORETA) [13], standardized LORETA (sLORETA) [14], and local autoregressive average (LAURA) [15]. These methods are computationally efficient and provide distributed solutions that do not require prior assumptions about the number of sources. However, these methods typically favor spatially widespread solutions and tend to blur focal sources due to the ill-posed nature of the inverse problem and the regularization constraints. As a result, despite their widespread use, these methods often struggle to accurately localize sparse or focal sources.

Dipole fitting methods, in contrast, avoid the ill-posedness of the inverse problem by identifying a small set of equivalent current dipoles (ECDs) whose fields best match the M/EEG measurements using a least-squares approach [16]. Rather than assigning a current value to every possible dipole across a densely sampled source space, these methods concentrate on pinpointing specific focal sources. Key dipole fitting methods include beamformers [17], [18] and MUSIC [16], as well as recursive extensions such as RAP-MUSIC [19], Truncated RAP-MUSIC [20], and RAP Beamformer [21]. While recursive methods generally outperform non-recursive versions, they face limitations including a dependency on high signal-to-noise ratio (SNR) and the risk of canceling correlated sources. Recent advancements, such as the Alternating Projections (AP) method [22], [23] and DS-MUSIC [21], have addressed some of these challenges. In particular, the AP method has demonstrated reduced localization errors compared to RAP-MUSIC, especially in situations with highly correlated sources or low SNR, making it a highly effective option for real-world applications [22].

Although AP performs well in low SNR and high-correlation settings, it struggles to accurately model sources with spatial extent, as such sources cannot be adequately represented by single dipoles. To address this, the FLEX-AP method [24], [25] was recently developed to extend AP by estimating both focal and extended sources through progressively smoothed matrices for source patches of increasing extent. FLEX-AP, however, assumes full coherence (rank-1) for all components of an extended source, which does not always hold, as cortical patch activities often display varying degrees of coherence and may deviate from a rank1 model. In summary, current methods tend to favor either discrete or distributed sources, and even when extended sources are addressed, complete coherence is generally assumed—an assumption that may not reflect realistic neural activity.

To overcome these limitations, we introduce PATCH-AP, an enhanced version of the AP method designed to localize both discrete and extended neural sources. PATCH-AP bridges the simplicity of dipole fitting with the comprehensiveness of distributed source imaging by effectively modeling and localizing both rank-1 focal sources with no spatial extent as well as rank-2 extended patches. It leverages the data covariance matrix directly, rather than estimating the signal subspace, which improves robustness when localizing highly correlated sources. Additionally, it incorporates iterative refinement of candidate dipole locations following the initial search to improve source estimation accuracy. PATCH-AP was evaluated against existing methods, including distributed source imaging (MNE, sLORETA), dipole fitting methods (AP), and recent advances in extended source localization (Convexity-Champagne (CC) and FLEX-AP).

This study introduces the PATCH-AP method and evaluates its source localization performance relative to existing methods. Section II presents the problem formulation for localizing spatially smooth source signals, followed by the approach for introducing a rank-r approximation to model activation time courses. Section III details the computation of the PATCH-AP inverse solution. Section IV describes the methodology for estimating the amplitudes of the source patches. Results from simulated and real EEG data evaluations are provided in Section V. A discussion of the findings and limitations of the current work is presented in Section VI. The article concludes with a discussion in Section VII. Our findings demonstrate that PATCH-AP improves extended neural source localization accuracy, holding significant promise for advancing clinical and research applications in neuroscience.

## II. Materials & Methods

### A. Background

The electrical currents generated by the apical dendrites of pyramidal neurons in the cerebral cortex are generally considered the primary source of MEG and EEG signals [9], [26]. Clusters of thousands of pyramidal cortical neurons that are activated simultaneously can be represented as an equivalent current dipole (ECD), which serves as the fundamental unit for modeling neural activity in MEG and EEG localization techniques.

In M/EEG recordings, the data $y(t)\in R^{m}$ captured by m sensors at a given time t can be expressed as the sum of responses from k current dipoles distributed across the source space. This relationship is mathematically represented as:

(1) $$y(t)=\sum_{i=1}^{k}a\left(p_{i}\right)s_{i}(t)+n(t),$$

where $a\left(p_{i}\right)$ denotes the forward vector for the ith source at position $p_{i},\;s_{i}(t)$ represents the source activity at time t, and $n(t)\in R^{m}$ represents additive white Gaussian noise added at the sensor level to mimic realistic EEG/MEG conditions. The noise was modeled as independent and identically distributed (i.i.d.) Gaussian noise. The forward vector $a\left(p_{i}\right)$ is defined as:

(2) $$a\left(p_{i}\right)=L\left(p_{i}\right)q,$$

where $L\left(p_{i}\right)$ is the m×3 forward matrix at location $p_{i}$, and q is the 3×1 orientation vector of the ECD source. Depending on the application, the dipole orientation q may be fixed (known) or freely oriented (unknown and to be estimated) [3], [16].

Assuming that recordings are obtained at T discrete time points t∈[1,…,T], the matrix Y representing the sampled signals can be formulated as:

(3) $$[Y{]}_{m\times T}=[A{]}_{m\times k}[S{]}_{k\times T}+[N{]}_{m\times T},$$

where Y is the m×T EEG/MEG data matrix:

(4) Y=[y(1),⋯,y(T)],

A is the m×k forward mapping matrix at the k candidate dipole locations comprising the entire source space:

(5) $$A=\left[a\left(p_{1}\right),\cdots ,a\left(p_{k}\right)\right],$$

S is the k×T source time series matrix:

(6) $$S=\left[s\left(1\right),\cdots ,s\left(T\right)\right],$$

with $s(t)={\left[s_{1}(t),\cdots ,s_{k}(t)\right]}^{T}$, and N is the m×T noise data matrix:

(7) $$N=\left[n\left(1\right),\cdots ,n\left(T\right)\right].$$

In this work, we restrict analyses to *fixed-oriented* dipoles, which align with the collective orientation of pyramidal cell dendrites [9], [16], [26]. Traditional dipole fitting methods, such as MUSIC, RAP-MUSIC, and AP, estimate the positions of N focal active sources from a set of k dipoles representing the entire source space. These methods assume that source signals (S) arise from rank-1 dipole sources and do not account for extended sources. To address this limitation, the FLEX method [25] was developed to model extended brain sources, though it still assumes rank-1 sources.

Here, we propose the PATCH-AP method, which estimates extended sources, or “patches,” with a rank greater than 1, in contrast to prior methods that assume rank-1 sources. Notably, localizing both the positions and spatial extent of sources in patch models pose an inherently ill-posed inverse problem, as the number of unknown parameters (location and extent) exceeds the number of sensors available in EEG or MEG setups. To manage this complexity, we introduce source priors to constrain the source space: (1) spatial smoothness of sources, and (2) activation time courses with reduced rank.

### B. Spatially Smooth Source Signals

This section addresses the spatial smoothing of source signals, an essential property to account for correlations between neighboring dipoles within source patches. Let i∈[1,…,N] denote the index of the source patches. The set $P=\left\{p_{1},\cdots ,p_{N}\right\}$ represents the seed dipole (or center) locations of the source patches, and $L=\left\{l_{1},\cdots ,l_{N}\right\}$ denotes the corresponding source extents. The source extent $l_{i}$ is an integer that defines the order of the patch extent, indicating the inclusion of neighboring dipoles up to a order of $l_{i}$ from the center of the patch. For the ith patch, with location $p_{i}$ and extent $l_{i}$, the number of dipoles in the patch is given by $d_{i}$. The total number of dipoles across all patches is then $d=d_{1}+d_{2}+\ldots +d_{N}$.

To apply spatial smoothing to the source signals $\dot{S}$ (of dimension d×T), we introduce a smoothing operator K. This operator K, which will rely on the Laplacian operator, is designed to smooth the signals across the cortical mesh, effectively incorporating the spatial structure of the sources within the model.

#### 1) Laplacian Operator on Cortical Mesh:

The cortical mesh is typically represented as a triangulated surface that spans the entire source space. Spatial relationships between sources are captured using an adjacency matrix $𝒜\in R^{k\times k}$, where k denotes the number of grid points sampling the surface. This matrix encodes the spatial proximity of dipoles. Specifically, 𝒜 is defined as:

$${𝒜}_{i,j}=\left\{\begin{array}{ll} 1 & \;\text{if}\;\text{dipoles}\;i\text{and}\;j\text{are}\;\text{neighbors},\;\\ 0 & \;\text{if}\;\text{dipoles}\;i\text{and}\;j\text{are}\;\text{not}\;\text{neighbors}. \end{array}\right.$$

The adjacency matrix thus identifies which dipoles (sources) are spatially adjacent, allowing neighboring dipoles to influence each other’s signals during smoothing.

The degree matrix D is defined as a diagonal matrix, where each diagonal element $D_{ii}$ is the sum of the elements in the ith row of the adjacency matrix:

(8) $$D_{ii}=\sum_{j}{𝒜}_{ij}.$$

Using the above, the Laplacian matrix ℒ is then computed as:

(9) ℒ=D-𝒜,

The Laplacian matrix captures local variations in the local neighborhood on the grid and plays a central role in modeling spatial smoothness on the cortex.

#### 2) Smoothing Operator:

The smoothing operator K is constructed using the Laplacian matrix and is defined as:

(10) K=I-αℒ,

where 0<α<1 is a diffusion parameter that controls the degree of smoothing, and I is the identity matrix of size k×k. The smoothing operator K applies a diffusion-like process that penalizes large differences between neighboring dipoles, encouraging smoother spatial transitions across the patch.

#### 3) Patch Basis Matrix:

We define the patch basis matrix $E\in R^{k\times d}$ to represent the sources associated with each patch in the model. For each patch i, let $O_{i}=\left\{i_{1},i_{2},\ldots ,i_{d_{i}}\right\}$ denote the set of indices corresponding to the sources within that patch. The vector $e_{i_{j}}$ is a standard basis vector of dimension k×1, with a value of 1 at the index $i_{j}\in O_{i}$ and 0 elsewhere. Using these vectors, we construct the patch basis matrix:

(11) $$E=\left[E_{1},E_{2},\ldots ,E_{N}\right],$$

(12) $$E_{i}=\left[e_{i_{1}},e_{i_{2}},\ldots ,e_{i_{d}}\right].$$

where $d_{i}$ is the number of sources in patch i, and N is the total number of patches [27].

#### 4) Constructing Spatially Smooth Source Signals:

Finally, the spatially smooth source signals S are constructed by utilizing the above smoothing operator K with smoothness order q and the patch basis matrix E. Here, $K^{q}$ denotes the matrix K raised to the power q. The parameter q is an integer that determines the level of spatial smoothness, where q=0 corresponds to no smoothing. To facilitate comprehension, we mark the dimensions of the individual elements:

(13) $$[S{]}_{k\times T}={\left[K^{q}\right]}_{k\times k}[E{]}_{k\times d}[\dot{S}{]}_{d\times T}.$$

Using this, we can rewrite (3) as:

(14) $$[Y{]}_{m\times T}=[G(P,L){]}_{m\times d}[\dot{S}{]}_{d\times T}+[N{]}_{m\times T},$$

where

(15) $$G\left(P,L\right)=AK^{q}E,$$

(16) $$G\left(P,L\right)=\left[g\left(p_{i},l_{i}\right),\cdots ,g\left(p_{N},l_{N}\right)\right],$$

(17) $$g\left(p_{i},l_{i}\right)=AK^{q}E_{i}.$$

### C. Activation Time Courses of Reduced Rank

To make the source localization solution tractable, we approximate the forward model using a rank-r approximation. In the previous section, we described the forward model as:

(18) $$Y=Y_{1}+Y_{2}+\cdots +Y_{N}$$

(19) $$=\left[g\left(p_{1},l_{1}\right),g\left(p_{2},l_{2}\right),\ldots ,g\left(p_{N},l_{N}\right)\right]\left[\begin{array}{c} {\dot{s}}_{1}\\ {\dot{s}}_{2}\\ \vdots \\ {\dot{s}}_{N} \end{array}\right]+N,$$

where $g\left(p_{i},l_{i}\right)$ represents the forward model for the ith source patch, and ${\dot{s}}_{i}$ the corresponding source signal.

To reduce dimensionality, we perform a Singular Value Decomposition (SVD) of the forward model $g\left(p_{i},l_{i}\right)$ and approximate it with a rank-r approximation. The forward matrix for each source $g\left(p_{i},l_{i}\right)$ is decomposed as follows:

(20) $${\left[g\left(p_{i},l_{i}\right)\right]}_{m\times d_{i}}={\left[U_{i}\right]}_{m\times d_{i}}{\left[\sigma_{i}\right]}_{d_{i}\times d_{i}}{\left[V_{i}\right]}_{d_{i}\times d_{i}}^{T},$$

To approximate this matrix with reduced rank $r_{i}$ (where $r_{i}\leq d_{i}$), we keep only the first $r_{i}$ singular values and their corresponding singular vectors. This yields the rank-r approximation:

(21) $$\left[g\left(p_{i},l_{i}\right)\right]\approx \left[U_{\left\{p_{i},l_{i},r_{i}\right\}}\right]\left[\sigma_{\left\{p_{i},l_{i},r_{i}\right\}}\right]{\left[V_{\left\{p_{i},l_{i},r_{i}\right\}}\right]}^{T},$$

where:

- $U_{\left\{p_{i},l_{i},r_{i}\right\}}=U_{i}\left[:,1:r_{i}\right]$ is the matrix of the first $r_{i}$ left singular vectors,
- $\sigma_{\left\{p_{i},l_{i},r_{i}\right\}}=\sigma_{i}\left[:,r_{i}:r_{i}\right]$ is the matrix of the first $r_{i}$ singular values,
- $V_{\left\{p_{i},l_{i},r_{i}\right\}}=V_{i}\left[1:r_{i},:\right]$ is the matrix of the first $r_{i}$ right singular vectors.

Using the rank-r approximation of the forward matrix, we can rewrite the contribution of each source patch $Y_{i}$ as:

(22) $$Y_{i}={\left[U_{\left\{p_{i},l_{i},r_{i}\right\}}\right]}_{m\times r_{i}}{\left[{\ddot{S}}_{i}\right]}_{r_{i}\times T}$$

where ${\ddot{s}}_{i}$ represents the reduced-rank approximation of the activation time courses for the ith patch.

In summary, existing methods either rely on focal dipole models or assume full coherence across extended patches, which may not fully reflect realistic neural activity. To flexibly capture both focal and extended sources without imposing full coherence, PATCH-AP first applies a spatial smoothing operator K, which distributes activity smoothly across neighboring dipoles within each candidate patch. Following smoothing, the leadfield matrix corresponding to each patch may still exhibit internal redundancy due to correlations among nearby dipoles. PATCH-AP therefore applies a low-rank approximation using SVD, retaining the top r=2 singular vectors. The combination of smoothing and rank-2 approximation allows PATCH-AP to robustly handle both focal and spatially extended sources with varying coherence structures.

### D. Problem Formulation

In this section, we formulate the source localization problem, which aims to estimate the locations and extents of brain sources, as well as the corresponding source signals. The total signal recorded at m sensors can be written, utilizing the rank-reduced formulation from (22), as follows:

(23) $$Y=\sum_{i=1}^{N}\;{\left[U_{\left\{p_{i},l_{i},r_{i}\right\}}\right]}_{m\times r_{i}}{\left[{\ddot{s}}_{i}\right]}_{r_{i}\times T}+N$$

(24) $$=[U(P,L,R){]}_{m\times r}[\ddot{S}{]}_{r\times T}+N,$$

where $P=\left\{p_{1},\cdots ,p_{N}\right\}$ corresponds to the set of source locations, $L=\left\{l_{1},\cdots ,l_{N}\right\}$ is the set representing source extents, and $R=\left\{r_{1},\cdots ,r_{N}\right\}$ represents the set of ranks associated with each source. Here, $r=r_{1}+r_{2}+\cdots +r_{N}$ is the total rank of the system, combining contributions from all sources.

We define the objective function using a least-squares estimation criterion based on the observed sensor data Y. The problem can be stated as follows: Given the observed data Y, estimate the N source locations P, their extents L, ranks R, and source time courses $\ddot{S}$. The least-squares estimation criterion is:

(25) $$\{\overset{ˆ}{P},\overset{ˆ}{L},\overset{ˆ}{R},\overset{ˆ}{\dot{S}}\}=*{argmin}_{P,L,R,\ddot{S}}\|Y-U(P,L,R)\ddot{S}{\|}_{F}^{2},$$

where U(P,L,R) represents the forward matrix parameterized by source positions P, extents L, and ranks R, and $\ddot{S}$ are the reduced-rank source activation time courses. The objective is to minimize the Frobenius norm of the difference between the observed sensor data Y and the modeled sensor data $U(P,L,R)\ddot{S}$.

This problem is further refined by introducing an assumption about the patch rank. In (10), we defined a smoothing operator that generates a sequence of forward matrices with progressively increasing smoothness. This operator is given by:

(26) $$A_{q}=AK^{q},$$

where A is the original forward matrix, and $A_{q}$ is the smoothed forward matrix with smoothness order q. For each source patch, we compare the singular values of the original leadfield matrix to those of the smoothed leadfield matrix.

**Fig. 1.**
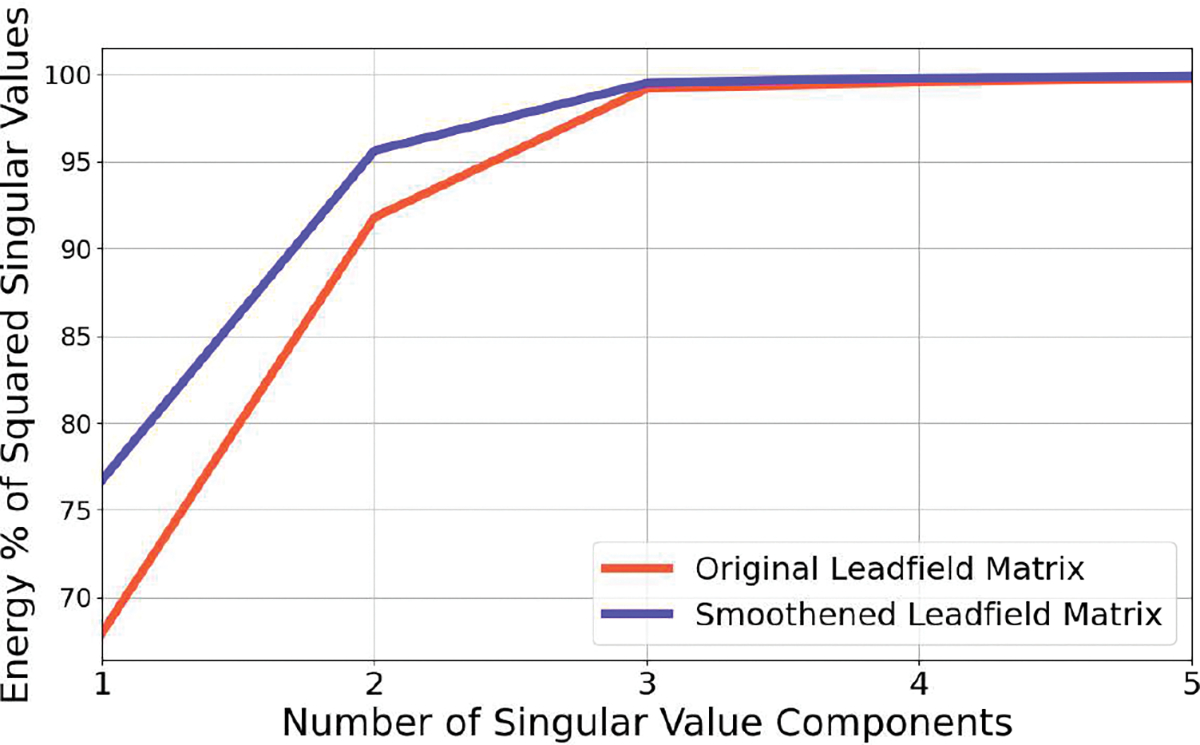
Energy Captured by Patch Activity of Different Ranks. Plot showing the cumulative energy percentage of squared singular values as a function of the number of singular value components for the original leadfield matrix (red) and the smoothed leadfield matrix (blue). The comparison highlights the effect of smoothing on the rank assumption.

Figure 1 shows the cumulative percentage of energy captured by the singular value components. Notably, for as few as two singular components, the cumulative energy of the smoothed leadfield matrix exceeds 95%, effectively capturing most of the variance. This observation is supported by extensive Monte Carlo simulations of 500 patches with extents l∈[0,…,5], where l=0 represents a point source and l=5 represents an extended source covering up to 5 neighboring dipoles (spanning 1.74 cm^2^ patch area on average). These simulations confirm that restricting the search to patches with a maximum rank of 2 is sufficient to capture the source contributions within each corresponding patch.

Consequently, substituting $R=\left\{r_{1}=2,\cdots ,r_{N}=2\right\}$ into (25), the objective function simplifies for the case of rank-2 sources:

(27) $$\{\overset{ˆ}{P},\overset{ˆ}{L},\overset{ˆ}{\dot{S}}\}=*{argmin}_{P,L,\ddot{S}}\|Y-U\left(P,L\right)\ddot{S}{\|}_{F}^{2}.$$

Thus, the estimated patch ranks are $\overset{ˆ}{R}=\left\{\overset{ˆ}{r_{1}}=2,\cdots ,\overset{ˆ}{r_{N}}=2\right\}$. It is important to note that the rank is related to the estimated source extent. Specifically:

- For a point source ($l_{i}=0$), the rank is $r_{i}=1$.
- For an extended source ($l_{i}>0$), the rank is set to $r_{i}=2$.

This rank assumption allows the model to account for additional degrees of freedom in the activity of extended sources with non-zero extents. By incorporating this information, we enhance the localization accuracy of sources with varying spatial extents, effectively capturing their activation dynamics.

## III. Patch-AP Inverse Solution

This section details the computation of the PATCH-AP inverse solution, which includes the standard Alternating Projections (AP) approach [22], [23] as a special case when the patch extent is set to 0 and the maximum rank is limited to 1. As noted previously, cortical patch activity is not fully coherent and does not always conform to rank 1. To address this limitation, we introduce an extension to the standard AP method, termed the PATCH-AP method.

In the earlier problem formulation section, the objective function for rank-2 sources was defined in (27). To simplify this further, we first express the unknown signal matrix $\ddot{S}$ in terms of the modified forward matrix U(P,L). This leads to the following solution for $\ddot{S}$:

(28) $$\overset{ˆ}{\ddot{S}}={\left(U(P,L{)}^{T}U(P,L)\right)}^{-1}U(P,L{)}^{T}Y,$$

Defining the projection matrix $\Pi_{U(P,L)}$ onto the column space of U(P,L) as:

(29) $$\Pi_{U(P,L)}=U(P,L){\left(U(P,L{)}^{T}U(P,L)\right)}^{-1}U(P,L{)}^{T},$$

we can rewrite the objective function (27) as:

(30) $$\{\overset{ˆ}{P},\overset{ˆ}{L}\}=*{argmin}_{P,L}{\left\|\left(I-\Pi_{U\left(P,L\right)}\right)Y\right\|}_{F}^{2},$$

(31) $$=*{argmax}_{P,L}{\left\|\Pi_{U(P,L)}Y\right\|}_{F}^{2}$$

(32) $$=*{argmax}_{P,L}tr\left(\Pi_{U(P,L)}C\right)$$

where tr() denotes the trace operator, $C={YY}^{T}$ is the data covariance matrix, and I is the identity matrix.

This formulation leads to a nonlinear and nonconvex N-dimensional maximization problem. The AP algorithm [28] addresses this by transforming the problem into a sequential, iterative process. During each iteration, the algorithm optimizes the parameters of a single source (location and extent) while holding the parameters of all other sources fixed at their previously estimated values.

Let j denote the current iteration and n the current source being estimated (with n=1 to N). During this process, the parameters of the other sources are fixed at their pre-estimated values. For sources 1 to (n-1), the parameters ${\left\{p_{i}^{j},l_{i}^{j}\right\}}_{i=1}^{n-1}$ have been pre-estimated in the current jth iteration. For sources n+1 to N, the parameters ${\left\{p_{i}^{j-1},l_{i}^{j-1}\right\}}_{i=n+1}^{N}$ were estimated in the previous j-1th iteration.

The matrix $A({\overset{ˆ}{P}}_{n}^{j},{\overset{ˆ}{L}}_{n}^{j})$ is defined as the m×(N-1) matrix of topographies corresponding to these parameter values (excluding the n-th topography):

(33) $$A\left({\overset{ˆ}{P}}_{n}^{j},{\overset{ˆ}{L}}_{n}^{j}\right)=\left[U_{\left\{p_{1}^{j},l_{1}^{j}\right\}},\ldots ,U_{\left\{p_{n-1}^{j},l_{n-1}^{j}\right\}},\right.\;\left.U_{\left\{p_{n+1}^{j-1},l_{n+1}^{j-1}\right\}},\ldots ,U_{\left\{p_{N}^{j-1},l_{N}^{j-1}\right\}}\right].$$

We decompose the projection matrix Π onto the column space of the augmented matrix $\left[A({\overset{ˆ}{P}}_{n}^{j},{\overset{ˆ}{L}}_{n}^{j}),U_{\left\{p_{n}^{j},l_{n}^{j}\right\}}\right]$ as:

(34) $$\begin{array}{l} \Pi_{[A({\overset{ˆ}{P}}_{n}^{j},{\overset{ˆ}{L}}_{n}^{j}),U_{\left\{p_{n}^{j},l_{n}^{j}\right\}}]}\;=\Pi_{A({\overset{ˆ}{P}}_{n}^{j},{\overset{ˆ}{L}}_{n}^{j})}+\Pi_{{\Pi^{⊥}}_{A({\overset{ˆ}{P}}_{n}^{j},{\overset{ˆ}{L}}_{n}^{j})}U_{\left\{p_{n}^{j},l_{n}^{j}\right\}}}\\ \;=\Pi_{A\left({\overset{ˆ}{P}}_{n}^{j},{\overset{ˆ}{L}}_{n}^{j}\right)}+\Pi_{Q_{n}^{j}U_{\left\{p_{n}^{j},l_{n}^{j}\right\}}}, \end{array}$$

where

(35) $$Q_{n}^{j}={\Pi^{⊥}}_{A({\overset{ˆ}{P}}_{n}^{j},{\overset{ˆ}{L}}_{n}^{j})},$$

is the projection matrix that removes contributions from all sources except the n-th source in the j-th iteration.

Substituting (34) into (32) and neglecting the first term (as it does not depend on $p_{n}$ and $l_{n}$), we finally obtain the PATCH-AP localizer function:

(36) $$\begin{array}{l} \left\{{\overset{ˆ}{p}}_{n}^{j},{\overset{ˆ}{l}}_{n}^{j}\right\}=\;*{argmax}_{p_{n},l_{n}}tr\left(\Pi_{Q_{n}^{j}U_{\left\{p_{n}^{j},l_{n}^{j}\right\}}}C\right)\\ \;=\;*{argmax}_{p_{n},l_{n}}tr\left(U_{\left\{p_{n}^{j},l_{n}^{j}\right\}}^{T}Q_{n}^{j}CQ_{n}^{j}U_{\left\{p_{n}^{j},l_{n}^{j}\right\}}\right.\\ \;\left.{\left(U_{\left\{p_{n}^{j},l_{n}^{j}\right\}}^{T}Q_{n}^{j}U_{\left\{p_{n}^{j},l_{n}^{j}\right\}}\right)}^{-1}\right). \end{array}$$

## IV. Patch Sources Amplitude Estimation

In this section, we describe the methodology for estimating the amplitudes of the source patches. For the i-th patch, the PATCH-AP method provides estimates of the patch’s location ${\overset{ˆ}{p}}_{i}$, extent ${\overset{ˆ}{l}}_{i}$, and rank ${\overset{ˆ}{r}}_{i}=2$. These parameters form the basis for estimating the source amplitudes $\overset{ˆ}{S}$ for each patch.

The first step is to compute $g({\overset{ˆ}{p}}_{i},{\overset{ˆ}{l}}_{i})$ based on these estimated parameters, and then apply SVD, decomposing it into orthogonal components:

(37) $${\left[g({\overset{ˆ}{p}}_{i},{\overset{ˆ}{l}}_{i})\right]}_{m\times d_{i}}={\left[{\overset{ˆ}{U}}_{i}\right]}_{m\times d_{i}}{\left[{\overset{ˆ}{\sigma }}_{i}\right]}_{d_{i}\times d_{i}}{\left[{\overset{ˆ}{V}}_{i}\right]}_{d_{i}\times d_{i}}^{T}$$

Here, ${\overset{ˆ}{U}}_{i}$ and ${\overset{ˆ}{V}}_{i}$ are orthogonal matrices representing the left and right singular vectors, respectively, and ${\overset{ˆ}{\sigma }}_{i}$ contains the singular values that describe the strength of the patch’s contribution to the sensor measurements. Since the estimated rank of each patch is ${\overset{ˆ}{r}}_{i}=2$, we approximate the contribution matrix $g({\overset{ˆ}{p}}_{i},{\overset{ˆ}{l}}_{i})$ by retaining the top ${\overset{ˆ}{r}}_{i}$ singular values. This rank reduction ensures computational feasibility while preserving the essential features of the patch’s contribution:

(38) $${\left[g({\overset{ˆ}{p}}_{i},{\overset{ˆ}{l}}_{i})\right]}_{m\times d_{i}}\approx [{\overset{ˆ}{U}}_{\left\{{\overset{ˆ}{p}}_{i},{\overset{ˆ}{l}}_{i},{\overset{ˆ}{r}}_{i}\right\}}][{\overset{ˆ}{\sigma }}_{\left\{{\overset{ˆ}{p}}_{i},{\overset{ˆ}{l}}_{i},{\overset{ˆ}{r}}_{i}\right\}}]{\left[{\overset{ˆ}{V}}_{\left\{{\overset{ˆ}{p}}_{i},{\overset{ˆ}{l}}_{i},{\overset{ˆ}{r}}_{i}\right\}}\right]}^{T}$$

To estimate the source amplitudes $\overset{ˆ}{\ddot{S}}$ across all patches, we use a minimum-norm estimate (MNE) framework. For the N detected sources, with their estimated locations $\overset{ˆ}{P}={\overset{ˆ}{p}}_{1},\ldots ,{\overset{ˆ}{p}}_{N}$, extents $\overset{ˆ}{L}={\overset{ˆ}{l}}_{1},\ldots ,{\overset{ˆ}{l}}_{N}$, and ranks $\overset{ˆ}{R}={\overset{ˆ}{r}}_{1},\ldots ,{\overset{ˆ}{r}}_{N}$, we form the combined left singular vector:

(39) $$U(\overset{ˆ}{P},\overset{ˆ}{L},\overset{ˆ}{R})=\left[{\overset{ˆ}{U}}_{\left\{{\overset{ˆ}{p}}_{1},{\overset{ˆ}{l}}_{1},{\overset{ˆ}{r}}_{1}\right\}},\ldots ,{\overset{ˆ}{U}}_{\left\{{\overset{ˆ}{p}}_{N},{\overset{ˆ}{l}}_{N},{\overset{ˆ}{r}}_{N}\right\}}\right].$$

Since we assumed uniform rank, this simplifies to $U(\overset{ˆ}{P},\overset{ˆ}{L},\overset{ˆ}{R})=U(\overset{ˆ}{P},\overset{ˆ}{L})$. The amplitude of the sources within each patch can now be reconstructed by computing:

(40) $$\overset{ˆ}{\ddot{S}}={\left(U^{T}(\overset{ˆ}{P},\overset{ˆ}{L})U(\overset{ˆ}{P},\overset{ˆ}{L})\right)}^{-1}U^{T}(\overset{ˆ}{P},\overset{ˆ}{L})Y$$

(41) $$\overset{ˆ}{\ddot{S}}=\left[\begin{array}{c} {\overset{ˆ}{\ddot{S}}}_{1}\\ \vdots \\ {\overset{ˆ}{\ddot{S}}}_{N} \end{array}\right]$$

The source time course ${\overset{ˆ}{\dot{s}}}_{i}$ for each patch can be estimated using:

(42) $${\overset{ˆ}{\dot{s}}}_{i}={\left\{\left[{\overset{ˆ}{\sigma }}_{\left\{{\overset{ˆ}{p}}_{i},{\overset{ˆ}{l}}_{i},{\overset{ˆ}{r}}_{i}\right\}}\right]{\left[{\overset{ˆ}{V}}_{\left\{{\overset{ˆ}{p}}_{i},{\overset{ˆ}{l}}_{i},{\overset{ˆ}{r}}_{i}\right\}}\right]}^{T}\right\}}^{†}{\overset{ˆ}{\ddot{S}}}_{1}$$

where † denotes the Moore-Penrose pseudoinverse. The overall source time courses for all patches can be combined into:

(43) $$\overset{ˆ}{\dot{S}}=\left[\begin{array}{c} {\overset{ˆ}{\dot{S}}}_{1}\\ \vdots \\ {\overset{ˆ}{\dot{S}}}_{N} \end{array}\right]$$

To achieve spatially smooth sources, the estimated source currents can be smoothed using a rank-specific spatial smoothing operator $K^{{\overset{ˆ}{r}}_{i}}$ and estimated patch basis functions $\overset{ˆ}{E}$ as follows:

(44) $$[\overset{ˆ}{S}{]}_{k\times T}={\left[K^{{\overset{ˆ}{r}}_{i}}\right]}_{k\times k}[\overset{ˆ}{E}{]}_{k\times d}[\overset{ˆ}{\dot{S}}{]}_{d\times T}$$

The process described ensures that the estimated source currents are not only rank-reduced but also spatially smooth and coherent with the underlying physical structure of the sources, making it more consistent with the realistic assumptions about the spatial distribution of the sources.

To summarize the full PATCH-AP procedure, Algorithm 1 provides a step-by-step pseudocode description of the complete localization framework.

### A. Evaluation on Simulation

For our evaluation, we used an EEG setup with 128 channels arranged according to the International 10–5 electrode placement system. To avoid the issue known as the “inverse crime”—which occurs when simulated and inversion data share the same forward model assumptions—we employed distinct forward models for data synthesis and inversion. We adopted a lower resolution (5124 points) for the forward solution and a higher resolution (8196 points) for the inverse solution to remain consistent with our prior studies [22], [24], [25], [29], where this configuration was successfully used to mitigate the inverse crime [30], [31]. Each simulation employed a source space comprising 5,124 fixed-oriented dipoles distributed across a cortical surface mesh. Although we did not explicitly separate sources by cortical depth, the chosen source space, inherently including patches with a range of eccentricities. We set the conductivity ratio between the skull and brain to 1:80, using conductivities of 0.33 S/m^2^ for brain and scalp tissues and 0.0042 S/m^2^ for the skull [32]. The forward matrix was computed using the boundary element method (BEM) [33]. For the inverse modeling, we used the same forward solution but increased the spatial resolution to k=8,196 dipoles with octahedral spacing, while retaining all other parameters, including the skull-to-brain conductivity ratio of 1:80.


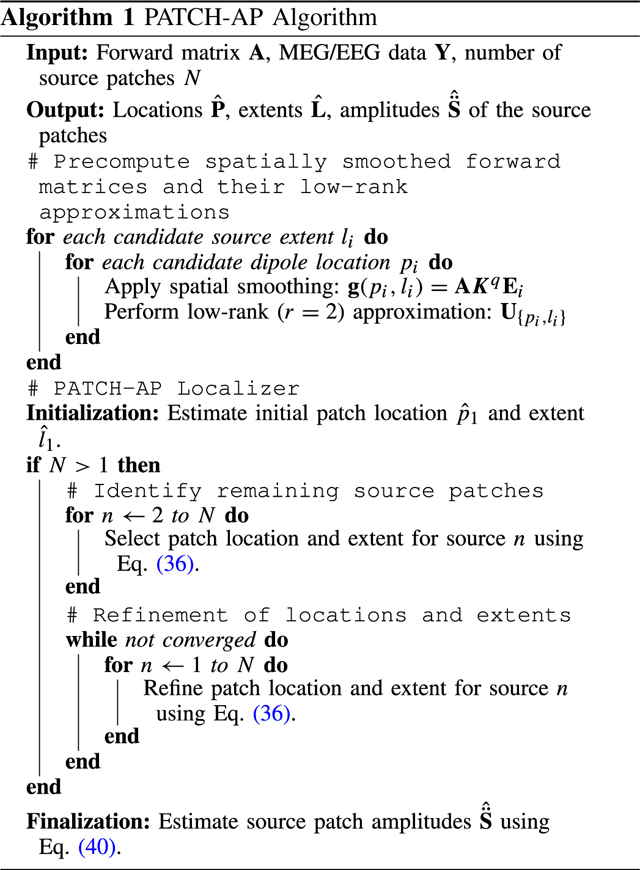


We conducted an experiment to illustrate source localization results in multi-source scenarios with distinct rank combinations, alongside three additional experiments, each varying a single parameter and simulating 500 Monte Carlo samples of neural activity projected onto 128 EEG channels. Experiment 1 investigated the effects of varying noise levels by altering the SNR to −5 dB, 0 dB, and 5 dB for a single patch of rank [2], combination of two patches with ranks [1, 2], and a combination of three patches with rank [1, 2, 2]. The inter-source correlation was set to ρ=0.5, and the smoothing parameter to 2. Experiment 2 examined the impact of inter-source correlation by adjusting ρ to {0.1, 0.5, 0.9} for patch ranks [2], [1, 2] and [1,2, 2]. We kept the SNR at 0 dB and used a smoothing parameter of 2. Experiment 3 analyzed the influence of smoothness order by varying it across 0, 2, 4 for patch ranks [2], [1, 2] and [1,2, 2], fixing the SNR at 0 dB, and the correlation coefficient at ρ=0.5. It is to note that the 500 Monte Carlo simulations were performed using independently and randomly selected patch locations for each configuration. For every simulation run, new patch centers were sampled, and the results were averaged across trials.

Inter-source correlation was introduced using a mixing matrix $C_{\mathrm{mix}}$, derived from Cholesky decomposition:

(45) $$\;C\;=1_{n\times n}\cdot \rho +I_{n}(1-\rho )\;C_{\text{mix}}\;=Cholesky(C)$$

where I is the identity matrix, ρ represents the desired correlation, and n is the number of sources. The matrix $C_{\text{mix}}$ was then used to mix the source time courses. After projecting the source vectors J through the forward matrix, independent identically-distributed noise was added to achieve an SNR of 0 dB.

We evaluated the performance of the Patch-AP method against its greedy counterparts (AP and FLEX-AP) as well as established methods such as MNE, sLORETA, and Convexity-Champagne. FLEX-AP was implemented following the approach outlined in Hecker et al. [25], [34]. All methods were implemented using the Python programming language. Specifically, AP, FLEX-AP, and CC were implemented using the open-source invert library,^1^ while MNE and sLORETA were implemented using the MNE-Python toolbox.

For the non-greedy methods, Tikhonov regularization was applied, with the optimal parameter selected from a set of ten options based on the generalized cross-validation criterion. For Convexity-Champagne, hyperparameters were tuned until the loss converged to a value below 10^−8^, using an active set strategy to eliminate near-zero candidates.

For the evaluation, we chose to use the Earth Mover’s Distance (EMD), also known as the Wasserstein metric. EMD quantifies the minimum effort required to transform one distribution into another, measured in millimeters based on the Euclidean distance between dipoles. The EMD calculations were performed using the Python POT package [35]. We selected EMD because it captures both spatial displacement and dispersion, which is essential when sources range from focal dipoles to extended, multi-component patches. Point-based metrics like Source Localization Error (SLE) rely on a single true and estimated peak location—an assumption that fails for rank-2 or higher patches, which lack a unique maximum intensity point. Consequently, EMD is our preferred metric for evaluating PATCH-AP across both focal and extended sources, as point-based measures such as SLE and intensity-error cannot accurately characterize multi-peak activation patches.

**Fig. 2.**
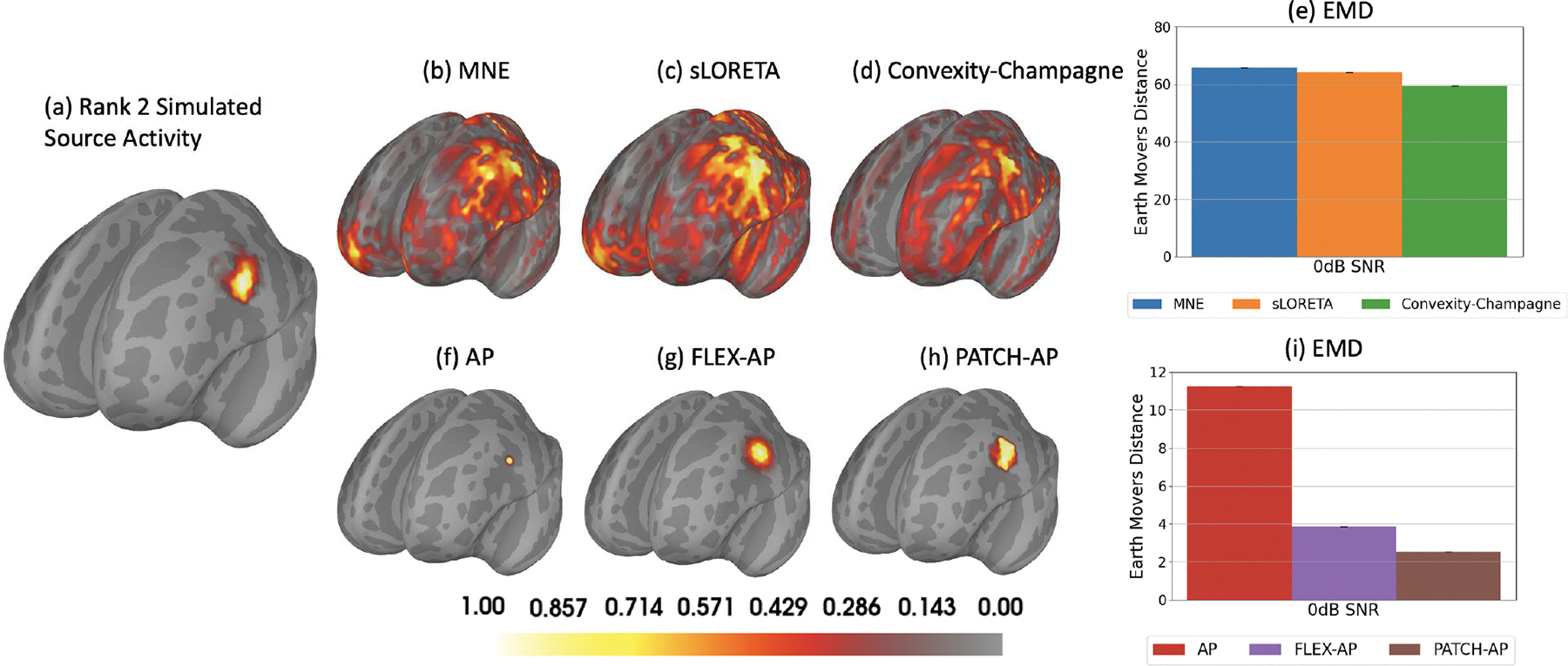
Localization of a single rank-2 extended source at 0 dB SNR. (a) Ground-truth source distribution. (b) MNE inverse solution. (c) sLORETA inverse solution. (d) Convexity-Champagne inverse solution. (e) Earth mover’s Distance (EMD) corresponding to (b), (c) and (d) inverse methods. (f) AP inverse solution. (g) FLEX-AP inverse solution. (h) Patch-AP inverse solution. (i) Earth mover’s Distance (EMD) corresponding to (f), (g) and (h) inverse methods.

Figure 2 illustrates the rank-2 extended simulated source activities and the corresponding inverse solutions. Panel (a) shows the simulation of a rank-2 source at 0 dB SNR. Panels (b), (c), and (d) display the inverse solutions obtained using the MNE, sLORETA, and CC methods, respectively. Panel (e) presents the EMD metrics for these three inverse methods. Panels (f), (g), and (h) depict the inverse solutions from the AP, FLEX-AP, and PATCH-AP methods, respectively, while panel (i) shows the EMD metrics corresponding to the inverse solutions from AP, FLEX-AP, and PATCH-AP. This figure highlights the limitations of dipole imaging and dipole fitting methods when applied to sources that are neither strictly focal nor extended across the entire cortical surface. Notably, PATCH-AP demonstrates the lowest EMD among all evaluated methods, indicating superior accuracy in source localization.

### B. Evaluation on Real Data

We evaluated the performance of the PATCH-AP algorithm on two real MEG datasets: (i) the SPM Face dataset, collected by R. Henson and available through the Statistical Parametric Mapping (SPM) toolbox,^2^ and (ii) the Brainstorm auditory dataset, available from the MNE-Python documentation^3^

#### 1) Spm Face Dataset:

This dataset consists of two sessions in which a subject views 86 face images and 86 scrambled face images. The MEG data were recorded on a 275-channel CTF/VSM system with second-order axial gradiometers and synthetic third gradient denoising, sampled at 480 Hz. Due to one faulty sensor, 274 MEG channels were used in this dataset. For our analysis, we evaluated both sessions of the MEG data; however, results from Session 2 are presented in this manuscript for brevity. The PATCH-AP method is compared against traditional techniques, including MNE, sLORETA, CC, AP, and FLEX-AP.

The MEG data were preprocessed prior to analysis. The raw MEG data were band-pass filtered with a high-frequency cutoff of 30 Hz using a finite impulse response (FIR) filter, designed with the “firwin” method, known for its flexibility and effectiveness in creating a windowed sinc FIR filter. This filtering step removes high-frequency noise and artifacts, retaining only the relevant frequency components for analysis. Next, the continuous MEG data were segmented into epochs to isolate neural responses related to specific events. A total of 168 epochs were extracted, each spanning from −0.2 seconds before to 0.6 seconds after the event onset. Baseline correction was applied using the interval from −0.2 to 0 seconds, normalizing the data by removing any DC offset present in the pre-stimulus period. These epochs were then averaged to produce evoked responses, and a contrast was computed between the two conditions: “Faces” versus “Scrambled Faces”.

After epoching, noise covariance was calculated using all 168 trials with the mne.compute_covariance function, focusing on the pre-stimulus period (tmax=0) to capture only noise-related activity. An empirical method was used for the covariance estimation, and no rank constraint was imposed (rank = None), allowing the full noise spectrum to be included in subsequent analyses. The covariance matrix was then applied to whiten the evoked response and transform the forward matrix accordingly. The source model comprised k=8196 vertices with fixed orientations along a cortical mesh.

#### 2) Brainstorm Auditory Dataset:

This dataset involves binaural auditory stimulation using intra-aural earphones. Data were recorded from a single subject using a 275-channel CTF MEG system at a sampling rate of 2400 Hz. Two acquisition runs of approximately six minutes each were concatenated for analysis. An empty-room recording was used to compute the noise covariance matrix, and preprocessing followed the recommended pipeline in MNE-Python. The continuous data were band-pass filtered and segmented into epochs around auditory stimulus events. Only epochs corresponding to the standard “beep” stimuli were retained for analysis. Because auditory evoked responses are typically bilateral, we assumed two active sources during inverse modeling.

## V. Results

We present four distinct experiments using simulated data to compare the accuracy of Patch-AP with other algorithms, evaluating performance under varying conditions: (A) Illustration in multi-source scenarios with distinct rank combination [1,3], Effect of (B) signal-to-noise ratios (SNRs), (C) inter-source correlation (ISC), (D) smoothness order, and (E) colored noise on inverse solution accuracy. Additionally, we analyze PATCH-AP inverse solutions applied to real SPM Faces dataset and brainstorm auditory dataset.

### A. Illustration in Multi-Source Scenarios With Distinct Rank Combinations

**Fig. 3.**
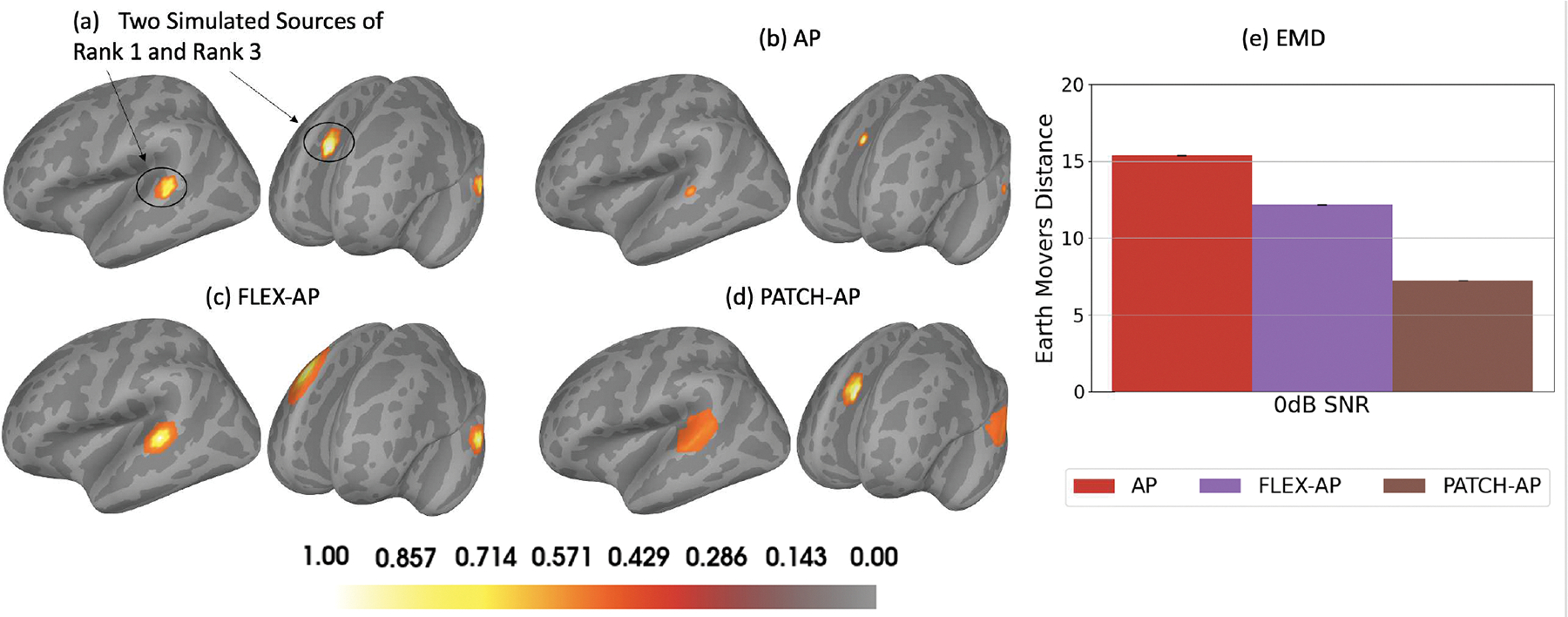
Localization performance in multi-source scenarios involving distinct patch ranks at 0 dB SNR, and inter-source correlation ρ=0.5. (a) Ground-truth configuration with one rank 1 source and one rank 3 source. (b) AP inverse solution. (c) FLEX-AP inverse solution. (d) Patch-AP inverse solution. (e) Earth mover’s Distance (EMD) corresponding to (b), (c) and (d) inverse methods.

In this evaluation, we illustrate the inverse solution of PATCH-AP in comparison to other methods for scenarios involving multiple sources with distinct rank combinations. Figure 3 (a) illustrates one rank 1 and one rank 3 extended simulated source activity at 0 dB SNR. Both sources have an extent of 2, spanning a 0.26 cm^2^ patch area on the cortex. Figure 3 (b), (c), and (d) display the inverse solutions obtained using AP, FLEX-AP, and PATCH-AP methods, respectively. Finally, 3 (e) shows the EMD metrics corresponding to the inverse solutions. The EMD results demonstrate the superior performance of the PATCH-AP method in accurately localizing sources in multi-source scenarios with distinct rank combinations, outperforming both the FLEX and AP methods. These findings emphasize the effectiveness of PATCH-AP in extended source localization and its potential for improved accuracy in complex source configurations.

### B. Effect of SNR

**Fig. 4.**
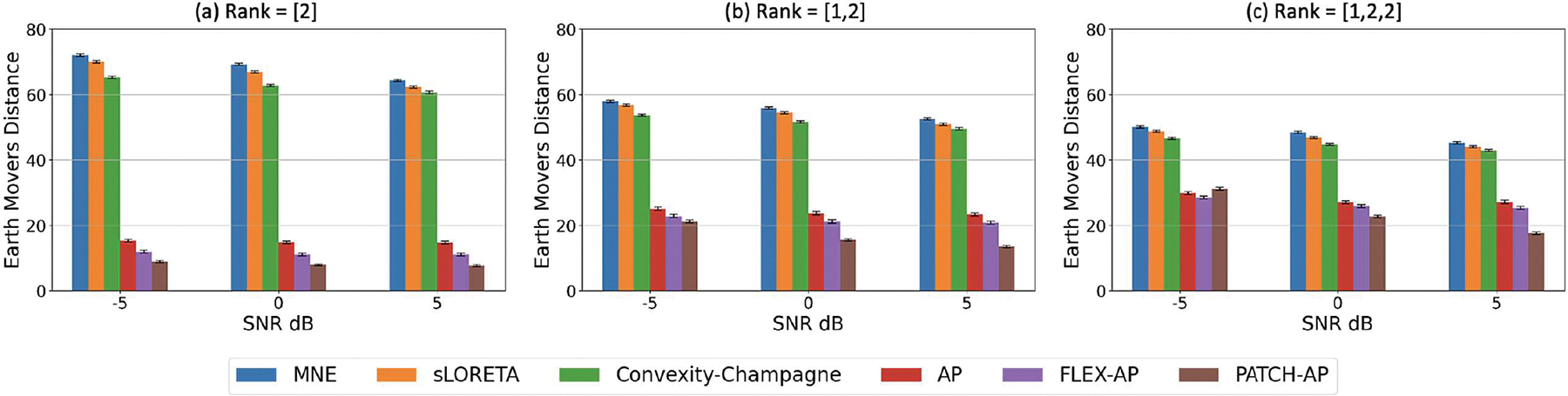
Effect of SNR on Inverse Solution Accuracy. Bar plots of the Earth Mover’s Distance (EMD) for various inverse solution methods at three SNR levels (−5 dB, 0 dB, and 5 dB), demonstrating the impact of noise on source localization accuracy. Results are presented for different source rank configurations: (a) Rank = [2], (b) Rank = [1, 2], and (c) Rank = [1, 2, 2]. The inter-source correlation coefficient is fixed at ρ=0.5, and the smoothing parameter set to 2. Lower EMD values indicate better localization performance, with the PATCH-AP method consistently showing superior results across all scenarios.

The simulation experiment assessed the performance of the inverse solution under varying noise levels, simulating realistic MEG recordings with SNRs ranging from −5 to 5 dB. The Earth Mover’s Distance (EMD) metric was computed for different source configurations: a single source with a rank of [2], two sources with a rank combination of [1,2], and three sources with a rank combination of [1,2,2]. Figure 4 shows that PATCH-AP consistently outperformed MNE, sLORETA, CC, AP, and FLEX-AP across all SNRs and ranks. Notably, AP, FLEX-AP, and PATCH-AP demonstrated significantly better performance than MNE, sLORETA, and CC. PATCH-AP showed a clear advantage over MNE, sLORETA, and CC, while maintaining competitive performance with AP and FLEX-AP. Overall, the improvement in localization accuracy, as measured by EMD, was most pronounced with PATCH-AP.

### C. Effect of ISC

**Fig. 5.**
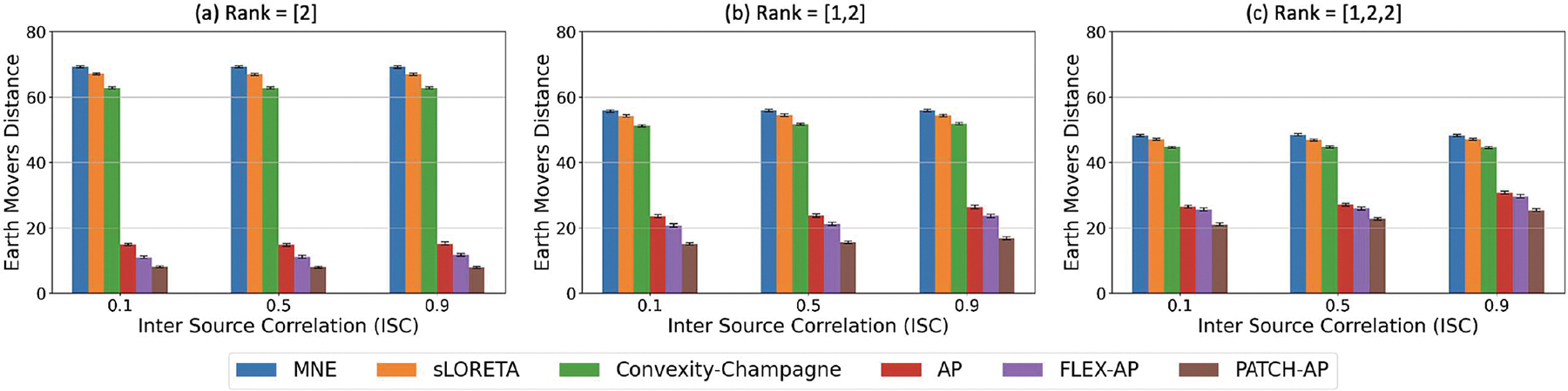
Effect of Inter-Source Correlation (ISC) on Inverse Solution Accuracy. Earth Mover’s Distance (EMD) for various inverse methods at different ISC levels (0.1, 0.5, 0.9), with results shown for rank configurations: (a) Rank = [2], (b) Rank = [1, 2], and (c) Rank = [1, 2, 2]. Simulations are performed at 0 dB SNR with a smoothing parameter of 2.

Accurately identifying underlying sources becomes increasingly challenging when they are highly correlated, highlighting the need for methods that can effectively differentiate sources in such scenarios. Figure 5 illustrates the accuracy of PATCH-AP across a range of inter-source correlations, from 0.1 (low correlation) to 1 (complete coherence). The simulations included different source configurations: a single source with a rank of [2], two sources with a rank combination of [1,2], and three sources with a rank combination of [1,2,2]. AP, FLEX-AP, and PATCH-AP consistently outperformed MNE, sLORETA, and CC under both low and high correlation conditions. Among all methods, PATCH-AP achieved the lowest EMD across various correlation levels, demonstrating its robustness in accurately localizing sources even in the presence of source correlations.

### D. Effect of Smoothness Order

**Fig. 6.**
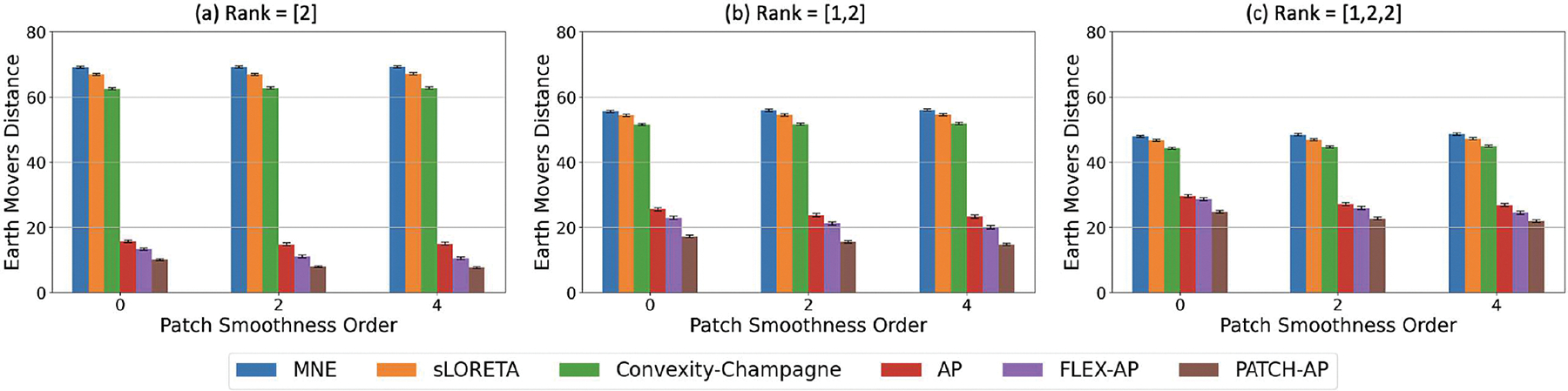
Effect of Patch Smoothness Order on Inverse Solution Accuracy. Earth Mover’s Distance (EMD) for various inverse methods across different patch smoothness orders (0, 2, 4). Results are shown for rank configurations: (a) Rank = [2], (b) Rank = [1, 2], and (c) Rank = [1, 2, 2], with simulations performed at 0 dB SNR and a correlation coefficient of ρ=0.5.

In our simulations, we analyzed the performance of source localization methods across different patch smoothness orders. Figure 6 illustrates the EMD metric as the smoothness order varies from 0 (no smoothness) to 4 (high smoothness). The simulations included several source configurations: a single source with a rank of [2], two sources with a rank combination of [1,2], and three sources with a rank combination of [1,2,2]. The results highlight the superior performance of PATCH-AP compared to all other methods across various smoothness orders.

### E. Effect of Colored Noise

To ensure that the simulated data closely resemble real-world conditions, we implemented multiple types of colored noise, following the approach in [29], each introducing noise covariance matrices with spatial correlations of varying structures and strengths across channels.

#### 1) Diagonal Noise:

This type of noise varies in power across channels while maintaining zero cross-channel correlation. In practice, we construct this matrix by alternately adding and subtracting a coefficient 0<γ<1 from the diagonal elements of the identity matrix. This results in non-uniform noise power across channels, with the standard deviation (SD) of the noise power across all channels equal to γ, which we refer to as the “SD of noise power.” Such variations in noise levels are commonly observed in real (non-whitened) data, where not all sensors are equally affected by noise.

#### 2) Banded Noise:

To model noise that spreads across neighboring sensors (e.g., muscle artifacts, blink artifacts, or head movements), we introduce correlations between adjacent channels. Each channel receives contributions from its immediate neighbors, scaled by $\gamma /\sqrt{2}$, where 0<γ<1 controls the correlation strength. This produces a banded structure in the covariance matrix, simulating realistic noise sources that affect multiple sensors simultaneously.

#### 3) Cholesky Noise:

This type introduces global correlations that decay exponentially with the distance between channels. The covariance matrix is defined as

$$C_{ij}=\gamma^{\left|i-j\right|},$$

where 0<γ<0.5 is the noise color coefficient that controls the correlation decay rate. Cholesky decomposition of this matrix is then applied to generate spatially colored noise, simulating widespread but gradually diminishing noise influences.

**Fig. 7.**
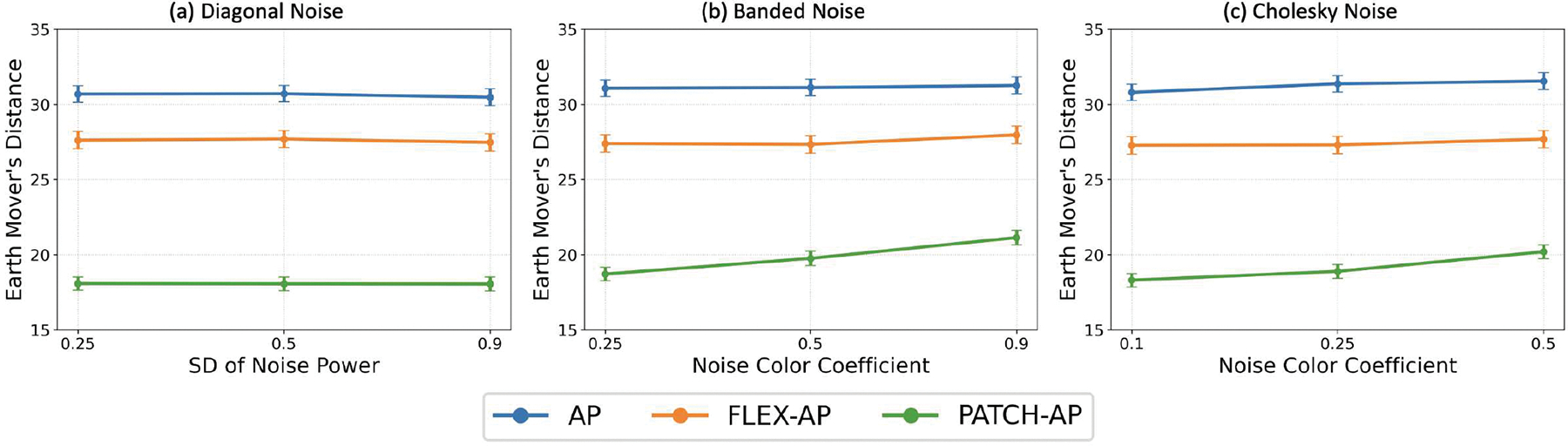
Effect of Colored Noise on Inverse Solution Accuracy. Comparison of AP, FLEX-AP, and PATCH-AP performance under different colored noise conditions: (a) Diagonal noise, (b) Banded noise, and (c) Cholesky noise. The experiments simulated realistic MEG recordings at an SNR of 0 dB. The Earth Mover’s Distance (EMD) was computed for [1,2] source configurations, with the inter-source correlation coefficient fixed at ρ=0.5 and the smoothing parameter set to 2.

These noise models, implemented in our simulation framework, enable the evaluation of algorithmic performance under both realistic and challenging noise conditions. The experiments simulated realistic MEG recordings at an SNR of 0 dB. The Earth Mover’s Distance (EMD) metric was computed for [1,2] source configurations, with the inter-source correlation coefficient fixed at ρ=0.5 and the smoothing parameter set to 2. As illustrated in Figure 7, PATCH-AP consistently outperformed AP and FLEX-AP across all spatial correlation levels and noise types.

### F. SPM Real Face Dataset

**Fig. 8.**
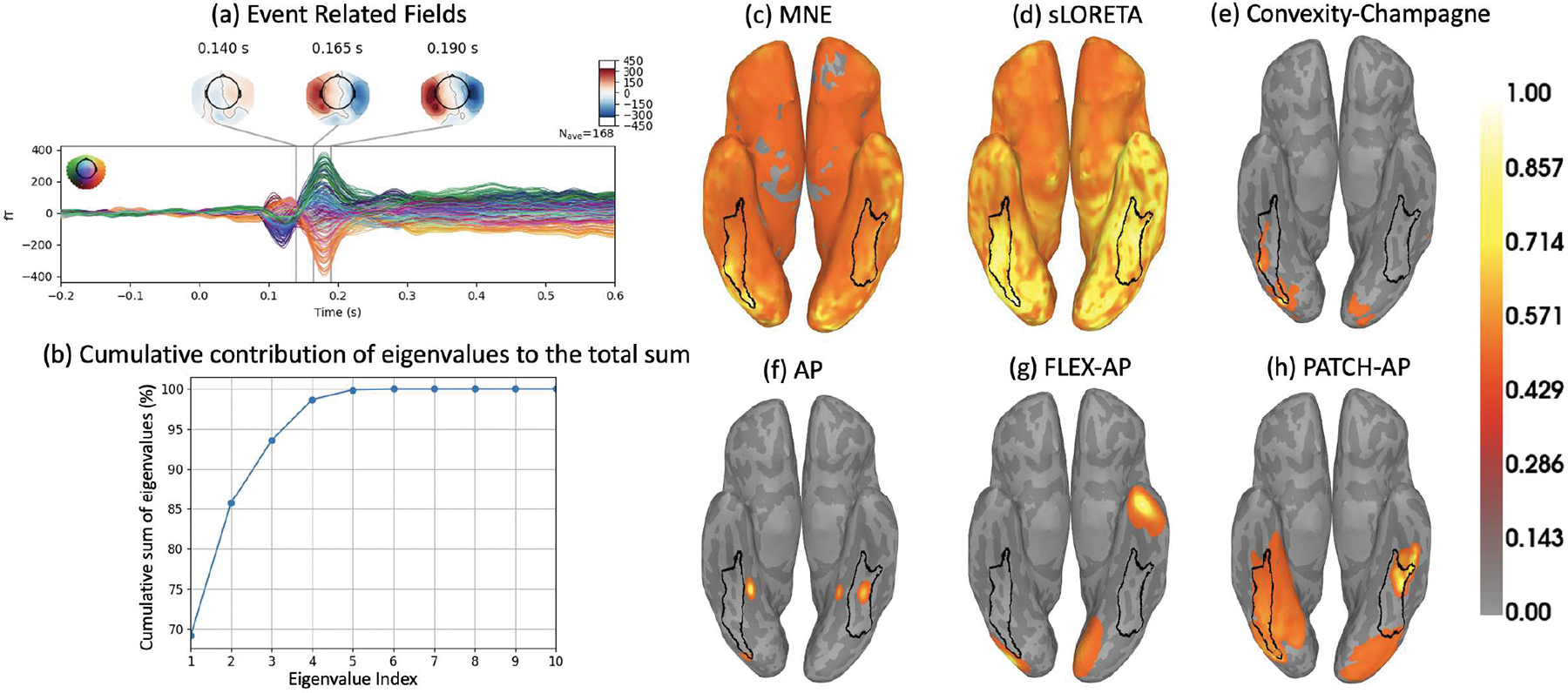
Source Localization on the SPM Faces Dataset. (a) Event-related fields (ERFs) in response to faces and scrambled objects, averaged over 168 trials and displayed as topographic maps of the magnetic fields in sensor space. (b) Cumulative contribution of eigenvalues to the total sum, showing the cumulative percentage of the total eigenvalue sum as a function of the eigenvalue index, indicating the number of sources required to capture significant variance. (c) MNE, (d) sLORETA, (e) Convexity-Champagne, (f) AP, (g) FLEX-AP, and (h) PATCH-AP inverse solutions. The PATCH-AP method demonstrates superior alignment in the fusiform face area (FFA), a region implicated in face processing, whereas AP and FLEX-AP exhibit lower localization accuracy.

Validating these algorithms on real data, however, is essential to confirm their practical applicability. In this study, we applied the source localization methods to human MEG data recorded during a face perception task. Figure 8(a) illustrates the event-related fields (ERFs) in response to a contrast between faces and scrambled faces, averaged over 168 clean epochs. Our analysis focused on the time window from 140 ms to 190 ms (25 time samples) to compute inverse solutions using PATCH-AP and other established algorithms.

To determine the number of sources in the real data, we assessed the ratio of the cumulative sum of eigenvalues to the total sum of the eigenvalues in the signal covariance matrix C. Figure 8(b) indicates the presence of four sources, as the cumulative sum surpasses 95% and eventually reaches 100%. Adding more sources beyond this point does not substantially increase the cumulative sum. This estimation is particularly important for the dipole fitting-based methods namely AP, FLEX-AP, and PATCH-AP, where specifying the number of sources is a required input to the dipole fitting procedure.

We then computed and visualized the source localization results, shown in Figures 8(c) through 8(h): (c) MNE, (d) sLORETA, (e) CC, (f) AP, (g) FLEX-AP, and (h) PATCH-AP. The proposed PATCH-AP method exhibits superior alignment with the fusiform face area (FFA), which is responsible for face processing, as highlighted by the black outline in the plot. In contrast, methods such as MNE, CC, AP, and FLEX-AP show less overlap with the FFA. Although sLORETA performs relatively well, showing an overlap comparable to PATCH-AP, the PATCH-AP method appears to offer the most accurate localization for both real and simulated data. It is worth noting that we analyzed both sessions of the MEG dataset; however, due to space constraints, only the results from Session 2 are presented in the manuscript. Session 1 analysis yielded consistent findings, further supporting the robustness of PATCH-AP.

### G. Brainstorm Auditory Dataset

**Fig. 9.**
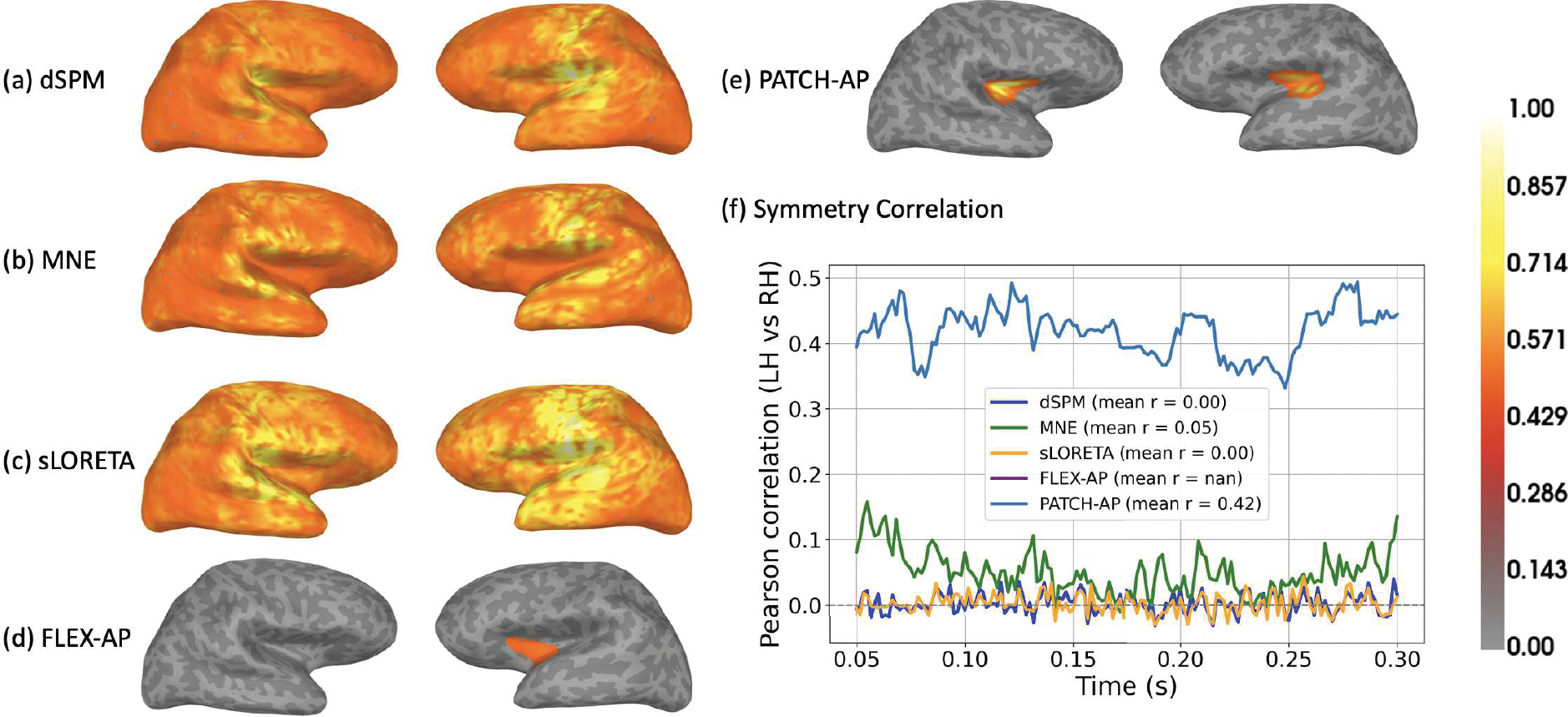
Source localization on the Brainstorm auditory dataset. Source estimates obtained using (a) dSPM, (b) MNE, (c) sLORETA, (d) FLEX-AP, and (e) PATCH-AP under regular beep auditory stimulation. PATCH-AP yields the most accurate bilateral activation in auditory cortex. (f) Symmetry correlation over time between left and right hemispheres. PATCH-AP achieves the highest mean correlation (ρ=0.42), indicating consistent bilateral localization.

To further assess the generalizability of the proposed PATCH-AP method across experimental paradigms, we performed an additional evaluation on the publicly available Brainstorm auditory MEG dataset.^4^ Figure 9 presents the inverse source localization results for (a) dSPM, (b) MNE, (c) sLORETA, (d) FLEX-AP, and (e) PATCH-AP. Among all methods, PATCH-AP successfully identified bilateral activation in the auditory cortex, demonstrating superior spatial precision. Notably, it localized the primary auditory sources without introducing spurious or non-auditory activations, in contrast to other techniques. AP failed to localize any auditory sources and is therefore not included in the figure.

The symmetry correlation coefficient is a valuable metric in MEG/EEG source analysis for quantifying the similarity of brain activity between the left and right hemispheres. It is particularly relevant for tasks such as auditory, language, or motor processing, where bilateral activation is expected. In our case, we employed this metric to assess the degree of bilateral activation. As shown in Figure 9(f), PATCH-AP achieved the highest symmetry correlation, with a mean ρ=0.42, indicating robust and symmetric source localization. In contrast, other methods yielded much lower correlation values, and FLEX-AP produced a NaN correlation because both sources were localized in the same (left) hemisphere, preventing bilateral comparison.

## VI. Discussion

The superior performance of PATCH-AP across both simulated and real MEG datasets suggests that incorporating spatial smoothness and low-rank structure offers meaningful advantages in accurately localizing complex neural sources. The lower EMD values indicate that PATCH-AP not only detects the correct regions of activation but also better preserves the spatial structure of extended sources. Traditional dipole-fitting methods such as AP and FLEX-AP aim to localize a small number of discrete sources and are well-suited to scenarios where neural activity is assumed to be focal. In contrast, distributed imaging methods such as MNE and sLORETA adopt a different philosophy: they regularize the inverse problem by minimizing the L2-norm of the source amplitudes, resulting in spatially diffuse activation maps. While this smoothness can be beneficial in suppressing noise and capturing broad activation patterns, it often comes at the expense of spatial precision and can blur distinct nearby sources.

PATCH-AP bridges the gap between these two classes of methods by explicitly modeling spatial smoothness in a structured way—via patch-based constraints—while also enforcing low-rank structure to capture complex activity within each patch. This enables PATCH-AP to recover sources that are neither overly focal (as in AP-type methods) nor overly smeared (as in MNE/sLORETA), but instead closely match the spatially extended and locally coherent patterns typically observed in real neural activity. We assessed PATCH-AP’s performance under varying conditions, including signal-to-noise ratios, inter-source correlations, patch smoothness orders, and colored noise. Additionally, we validated its applicability using real human MEG data from both a face perception task and an auditory evoked task.

In our evaluation, we strategically selected methods spanning a wide range of source localization paradigms—including dipole-fitting methods (AP, FLEX-AP), distributed imaging techniques (MNE, sLORETA), and the recent sparse Bayesian learning approach Convexity Champagne [36]—to ensure methodological diversity while remaining within the strict page limit. Additionally, in our recent work [24], we performed a comprehensive comparison among AP, FLEX-AP, RAP-MUSIC, and FLEX-RAP-MUSIC, in which AP and FLEX-AP consistently outperformed the RAP-based variants. Therefore, in the current manuscript, we focus on AP and FLEX-AP as the most competitive dipole-fitting baselines. Furthermore, we acknowledge the development of emerging deep-learning-based methods, such as 3D-PIUNet [37] and Multiple-Penalized State-Space (MPSS) models [38]. While these approaches represent promising directions, they rely heavily on task-specific data, hyperparameter tuning, and training pipelines, making their assumptions and evaluation strategies fundamentally different from the analytical, training-free design of PATCH-AP. For these reasons, we did not include direct comparisons with such methods. PATCH-AP addresses a long-standing methodological gap by explicitly modeling spatial smoothness and rank adaptivity, offering a complementary solution capable of localizing both focal and spatially extended sources.

The computational complexity of PATCH-AP is primarily determined by its iterative, greedy localization approach, which is structurally similar to the traditional Alternating Projection (AP) and FLEX-AP methods but involves additional preprocessing steps—namely, spatial smoothing and low rank approximations. These preprocessing steps incur a minor computational overhead and can be efficiently computed offline before the iterative localization process begins. Specifically, the complexity for spatial smoothing and low-rank approximations scales as O(k⋅m), where k is the number of candidate dipole locations, and m is the number of EEG/MEG sensors. Thus, when these steps are completed in advance, the real-time computational cost of PATCH-AP closely matches that of the traditional AP and FLEX-AP algorithms.

In the original AP article [22], the computational complexity was discussed, emphasizing that AP simplifies the multi-source localization problem into an iterative sequence of one-source maximization problems. A key benefit of the AP approach highlighted in [22] is its ability to avoid explicit computation of the signal subspace, unlike MUSIC-based methods. Instead, AP directly utilizes the data covariance matrix, allowing improved localization performance for highly correlated or coherent sources with computational costs comparable to other recursive scanning algorithms such as MUSIC or beamforming approaches. Indeed, the phantom experiments reported in [22] demonstrated that the AP algorithm typically converges within approximately three iterations, incurring computational costs similar to other recursive localization methods.

Beyond its methodological contributions, PATCH-AP has potential clinical relevance for applications involving neurological and psychiatric disorders where altered source extent or coherence is implicated. For example, in epilepsy, the ability to localize spatially extended epileptogenic zones may improve surgical planning. Similarly, in stroke, neurodegeneration, or cognitive impairment, detecting changes in cortical activation patterns across both focal and distributed regions may provide valuable biomarkers for diagnosis and monitoring. Furthermore, since PATCH-AP does not require data-driven training or prior patient-specific examples, it may be particularly well-suited for individualized clinical assessments in scenarios where patient data are limited.

A key limitation of PATCH-AP is its current restriction to modeling sources with rank ≤ 2. While this effectively captures many complex cortical activations, it may fail to fully reconstruct more complex or higher-rank source configurations, potentially leading to underestimation of spatial extent. In future work, we aim to extend the method to handle adaptive or higher-rank source modeling.

## VII. Conclusion

In summary, this study introduced PATCH-AP, an enhanced version of the Alternating Projection (AP) method, designed to overcome the limitations of traditional dipole fitting and distributed source imaging techniques in MEG source localization. PATCH-AP effectively localizes both discrete and extended sources, providing a more accurate representation of complex neural activity. Simulations demonstrated PATCH-AP’s robustness under varying SNR ratios, inter-source correlations, and patch smoothness conditions, consistently achieving lower EMD metrics compared to established methods like MNE, sLORETA, Convexity Champagne, AP, and FLEX-AP. Additionally, validation on real MEG data from a face perception task and auditory task highlighted PATCH-AP’s superior alignment with the fusiform face area (FFA) and auditory cortex region, confirming its practical utility in real-world applications. These results underscore PATCH-AP’s potential to significantly enhance clinical and research applications in neuroscience by enabling more precise and reliable neural source localization. Future research should continue to explore its efficacy across a broader range of experimental conditions and clinical scenarios, further establishing its value in neuroscience and clinical research.

## Data Availability

All code used to produce the results of this work can be found at https://github.com/Amita-Giri/PATCH-AP-Localization-of-Realistic-Spatial-Patches-of-Complex-Source-Activity-in-MEG-and-EEG

## Footnotes

1
https://github.com/LukeTheHecker/invert

2
https://www.fil.ion.ucl.ac.uk/spm/data/mmfaces/

3
https://mne.tools/stable/autotutorials/io/60_ctf_bst_auditory.html

4
https://mne.tools/stable/autotutorials/io/60_ctf_bst_auditory.html
